# Prevalence of Periodontal Disease and Periodontopathic Bacteria in Anti–Cyclic Citrullinated Protein Antibody–Positive At-Risk Adults Without Arthritis

**DOI:** 10.1001/jamanetworkopen.2019.5394

**Published:** 2019-06-07

**Authors:** Kulveer Mankia, Zijian Cheng, Thuy Do, Laura Hunt, Josephine Meade, Jing Kang, Val Clerehugh, Alastair Speirs, Aradhna Tugnait, Elizabeth M. A. Hensor, Jackie L. Nam, Deirdre A. Devine, Paul Emery

**Affiliations:** 1 Leeds Institute of Rheumatic and Musculoskeletal Medicine, University of Leeds, Leeds, United Kingdom; 2 National Institute for Health Research Leeds Biomedical Research Centre, Leeds Teaching Hospitals NHS Trust, Leeds, United Kingdom; 3 Division of Oral Biology, School of Dentistry, University of Leeds, Leeds, United Kingdom; 4 Leeds Dental Institute, Leeds Teaching Hospitals NHS Trust, Leeds, United Kingdom

## Abstract

**Importance:**

The prevalence of periodontitis is increased in patients with rheumatoid arthritis (RA) and periodontopathic bacteria can citrullinate proteins. Periodontitis may, therefore, be an initiator of RA and a target for prevention. Periodontal disease and periodontal bacteria have not been investigated in at-risk individuals with RA autoimmunity but no arthritis.

**Objective:**

To examine periodontal disease and periodontopathic bacteria in anti–cyclic citrullinated protein (anti-CCP) antibody–positive at-risk individuals without arthritis.

**Design, Setting, and Participants:**

This cross-sectional study took place at a teaching hospital from April 27, 2015, to May 8, 2017. Forty-eight anti-CCP–positive individuals without arthritis (CCP+ at-risk) were recruited nationally. Twenty-six patients with early RA (ERA) and 32 healthy control individuals were recruited locally. Data were analyzed between June 1, 2017, and December 1, 2017.

**Interventions:**

Periodontal assessment and examination of joints using ultrasonography.

**Main Outcomes and Measures:**

Prevalence of diseased periodontal sites, clinical periodontitis, and periodontal inflamed surface area in CCP+ at-risk individuals compared with patients with ERA and healthy individuals matched for age and smoking. Paired-end sequencing of DNA from subgingival plaque from diseased and healthy periodontal sites was performed and DNA was profiled and analyzed.

**Results:**

A total of 48 CCP+ at-risk individuals (mean [SD] age, 51.9 [11.4] years; 31 [65%] female), 26 patients with ERA (mean [SD] age, 54.4 [16.7] years; 14 [54%] female), and 32 healthy individuals (mean [SD] age, 49.4 [15.3] years; 19 [59%] female) were recruited. Of 48 CCP+ at-risk individuals, 46 had no joint inflammation on ultrasonography. Thirty-five CCP+ at-risk individuals (73%), 12 healthy individuals (38%), and 14 patients with ERA (54%) had clinical periodontitis. The median (interquartile range) percentage of periodontal sites with disease was greater in CCP+ at-risk individuals compared with healthy individuals (3.3% [0%-11.3%] vs 0% [0%-0.7%]) and similar to patients with ERA (1.1% [0%-13.1%]). Median (interquartile range) periodontal inflamed surface area was higher in CCP+ at-risk individuals compared with healthy individuals (221 mm^2^ [81-504 mm^2^] vs 40 mm^2^ [12-205 mm^2^]). Patients with CCP+ at-risk had increased relative abundance of *Porphyromonas gingivalis* (but not *Aggregatibacter actinomycetemcomitans*) at healthy periodontal sites compared with healthy individuals (effect size, 3.00; 95% CI, 1.71-4.29) and patients with ERA (effect size, 2.14; 95% CI, 0.77-3.52).

**Conclusions and Relevance:**

This study found increased prevalence of periodontitis and *P gingivalis* in CCP+ at-risk individuals. This suggests periodontitis and *P gingivalis* are associated with disease initiation and could be targets for preventive interventions in RA.

## Key Points

**Question:**

What is the prevalence of periodontal disease and citrullinating periodontopathic bacteria in anti–cyclic citrullinated protein–positive at-risk individuals (CCP+ at-risk) compared with a healthy control group and patients with early rheumatoid arthritis (RA)?

**Findings:**

This cross-sectional study identified an increased prevalence of periodontal disease sites, clinical periodontitis, and periodontal inflamed surface area in CCP+ at-risk individuals and those with early RA compared with a control group. Results showed that CCP+ at-risk individuals had increased abundance of *Porphyromonas gingivalis* at healthy periodontal sites compared with the control group and patients with early RA.

**Meaning:**

In individuals at risk of RA, periodontitis and *P gingivalis* were increased before joint disease and may be a target for prevention.

## Teaser

This cross-sectional study estimates the prevalence of periodontal disease and citrullinating periodontopathic bacteria in individuals who test positive for anti–cyclic citrullinated protein (anti-CCP) antibody without rheumatoid arthritis compared with groups of healthy control participants and patients with early rheumatoid arthritis.

## Introduction

Autoantibodies associated with rheumatoid arthritis (RA) can be detected in the serum years before patients develop joint inflammation,^1,2,3^ suggesting the joints may be a target rather than the primary cause of this disease. Such observations suggest a preclinical phase of RA and, importantly, raise the possibility of disease prevention. The enrichment of serum IgA anticitrullinated protein antibodies (ACPA) in individuals at risk of RA suggests mucosal sites (eg, oral mucosa) may be important in the earliest phase of RA.^4,5^ There is good evidence that periodontitis and RA are clinically associated.^6,7,8^ Furthermore, periodontitis is associated with a specific bacterial signature characterized by the increased abundance of the pathogenic organism *Porphyromonas gingivalis *alongside a community of other, predominantly anaerobic, organisms.^9^ *Porphyromonas gingivalis* is capable of citrullinating local antigens by virtue of its peptidylarginine deiminase enzyme.^10^ In a putative etiological model, virulent strains of *P gingivalis* at inflamed periodontal sites generate novel citrullinated antigens that trigger a mucosal immune response in certain individuals, possibly those with genetic predispositions.^11^ Recent data suggest the periodontopathic bacterium *Aggregatibacter actinomycetemcomitans* may also directly induce neutrophil citrullination at the periodontium^12^ and therefore potentially initiate ACPA.

Despite these observations, to our knowledge, periodontitis and citrullinating bacteria have not been described in individuals at risk of RA. We sought to comprehensively measure periodontitis and the abundance of key citrullinating bacteria in individuals who were ACPA positive (ie, individuals positive for anti–cyclic citrullinated protein [CCP] without synovitis and at risk of RA), patients with anti-CCP–positive early RA (ERA), and healthy control individuals. We hypothesized that (1) periodontitis would be similarly increased in CCP+ at-risk individuals and those with ERA compared with healthy individuals and (2) there would be an increased abundance of citrullinating periodontopathic bacteria in CCP+ at-risk individuals and patients with ERA compared with healthy individuals.

## Methods

### Design

A cross-sectional study of periodontal and clinical parameters was performed in CCP+ at-risk individuals, patients with ERA, and healthy individuals between April 27, 2015, and May 8, 2017. In this exploratory study, we aimed for 30 participants per group, in line with recommendations for pilot studies. Groups were approximately frequency matched during recruitment for age, sex, and smoking status. After 20 CCP+ at-risk individuals, 20 healthy individuals, and 10 patients with ERA were recruited, demographic and smoking data were reviewed. Approximate frequency matching was performed to recruit remaining healthy individuals and patients with ERA within the age range of 31 to 70 years and to recruit balanced numbers within the tertiles of age calculated in the first 20 CCP+ at-risk individual (first and third tertiles 52 and 60 years, respectively). We approximately matched the proportion of participants who had ever smoked, which was 60% in the first 20 CCP+ at-risk individuals.

A shotgun metagenomic analysis was performed on subgingival plaque samples collected during the periodontal assessments.

Ethical approval for this study was provided by the National Research Ethics Service Committee Yorkshire and the Humber, Leeds West. Written informed consent was received from all participants. This study followed the Strengthening the Reporting of Observational Studies in Epidemiology (STROBE) reporting guideline.^13^

### Clinical Participants

Anti-CCP–positive at-risk individuals with musculoskeletal symptoms but no clinical synovitis, patients with ERA, and asymptomatic healthy individuals were recruited at Chapel Allerton Hospital, Leeds, United Kingdom.

We recruited CCP+ at-risk participants from the Leeds at-risk cohort.^14,15^ Patients older than 18 years presenting to general practitioners or other health professionals with new-onset musculoskeletal symptoms but no clinical synovitis were invited to participate. Primary care recruitment was adopted nationally by the UK Primary Care Clinical Research Network. Anti-CCP testing was performed centrally using the Bioplex 2200 kit (BioRad). Those with a positive test result were invited to a dedicated research clinic in Leeds where recruitment for this study was undertaken. Patients from the Leeds early arthritis clinic who were anti-CCP–positive but did not have clinical synovitis were also recruited. Patients with ERA were all anti-CCP–positive and within the first 3 months of disease-modifying antirheumatic drug (DMARD) therapy. Control participants had no joint disease or history of inflammatory arthritis (and no affected first-degree relatives). Control individuals included coworkers at the University of Leeds (eg, academics, administrative workers, laboratory staff, cleaning staff) and people from the local community (eg, lay members of the Leeds Biomedical Research Centre Patient and Public Involvement group, their contacts, and local community groups). Control participants were typical of the general population with a range of socioeconomic groups represented.

Demographic details and a serum IgG anti-CCP2 test (Immunocap; Phadia) were taken at the time of the periodontal assessment.

### Periodontal Assessment

Periodontal assessments were performed at Chapel Allerton Hospital, Leeds. Periodontal status was assessed by 3 experienced dentists (V.C., A.S., and A.T.). Dentists were blinded to the RA status of the participants. A full-mouth examination of 6 sites on each natural tooth (recorded as a 6-point pocket chart) was performed on each participant. The 6 sites measured were the 4 corners of the tooth and the midpoint between the buccal and lingual aspects of the tooth. The following parameters were recorded at each available dental site: probing pocket depth (PPD) (millimeters), clinical attachment level (CAL) (millimeters), and presence of bleeding on probing (BOP) (indicated as present or absent). The PCP10 periodontal probe (Hu-Friedy) was used for assessment of BOP and PPD. Measurements of PPD were taken along the vertical axis of the tooth at each site using approximately 0.25 N of force.

Periodontal disease sites were determined according to the recent update to the 1999 American Academy of Periodontology Classification of Periodontal Diseases and Conditions.^16^ To ensure high sensitivity, thresholds for CAL and PPD were deliberately set so that sites with mild, moderate, and severe periodontitis would all be captured. Periodontal sites with 2 mm or greater CAL and 4 mm or greater PPD were defined as periodontitis sites (PDD) and considered to represent sites of current or past (including treated) periodontitis. Periodontal sites with 2 mm or greater CAL, 4 mm or greater PPD, and BOP were defined as active PDD and considered to represent current active periodontitis.

In addition to these parameters, dentists also classified all participants according to overall clinical periodontal status. The periodontal medical record for each participant was reviewed by the 3 dentists who were blinded to all patient details. In each case, clinical periodontal status was agreed by consensus and was classified as follows: (1) healthy (no periodontitis or gingivitis), (2) gingivitis only (no periodontitis), or (3) periodontitis with or without gingivitis. Classification was based on clinical judgment and the update to the 1999 American Academy of Periodontology Classification of Periodontal Diseases and Conditions,^16^ taking into account the distribution, extent, and severity of periodontitis and also the need for treatment.

### Periodontal Inflamed Surface Area

To quantify the total burden of periodontal inflammation, the total periodontal inflamed surface area (PISA) was calculated for each participant from PPD and CAL measurements at each dental site using the method described by Nesse et al.^17^ This index has been proposed as a way of more accurately quantifying inflamed periodontal tissues.^17^

### Ultrasonography Assessment

Ultrasonography (US) evaluation was performed on all CCP+ at-risk individuals by 2 experienced musculoskeletal sonographers (J.L.N. and L.H.). A standardized 38-joint US protocol was used (eAppendix 1 in the Supplement shows full details). Scans were performed using a Logiq E9 machine (General Electric) using a 15-6 MHz transducer. Power Doppler (PD) signal was assessed using a pulse repetition frequency set between 0.7 and 1.0 KHz, medium wall filter, and gain adjusted until background noise was suppressed. Doppler frequency was 10 MHz. Scoring of gray scale and PD synovitis was according to the European League Against Rheumatism Outcome Measures in Rheumatology scoring system.^18,19^

### Subgingival Plaque Collection

Healthy and diseased periodontal sites suitable for subgingival plaque collection were identified by dentists during the periodontal examination. Supragingival plaque was removed with cotton-wool pledgets prior to sample collection (eAppendix 2 in the Supplement).

### DNA Extraction, Library Preparation, and Sequencing

Subgingival plaque samples were thawed on ice from −80 °C. All diseased site samples were pooled and all healthy site samples were pooled for each participant. We extracted DNA from pooled samples using the UltraClean Microbial DNA Isolation Kit (Qiagen) as per manufacturer’s instructions and quantified by using PicoGreen dsDNA Reagent and Kits (Thermo Fisher Scientific) (eAppendix 2 in the Supplement).

The DNA was sheared to 200 base pairs (bp) in a small glass vial (microTUBE AFA Fiber Pre-Slit Snap-Cap 6 × 16 mm) by using a S220 Focused-ultrasonicator (Covaris). The size distribution of 4-fold diluted samples was evaluated on the Agilent 2200 TapeStation controlled by Agilent 2200 TapeStation Software A.01.05, using the Agilent High Sensitivity D1000 ScreenTape and Reagents.

Depending on the concentration of sheared DNA in the samples, either NEBNext Ultra DNA Library Prep Kit for Illumina or NEBNext Ultra II DNA Library Prep Kit for Illumina (New England Biolabs) was used for library construction, including end preparation, adaptor ligation, and polymerase chain reaction enrichment. AxyPrep Mag PCR Clean-up beads (Corning) were used for the clean-up steps during and after the library preparation to remove unincorporated adaptors, primers, adaptor dimers, primer dimers, and other contaminants. The size distribution and the quantity of the DNA libraries were checked using the method described. We pooled DNA libraries tagged with different index primers together and paired-end sequenced on the Hiseq 3000 machine (Illumina).

### Shotgun Metagenomic Data Processing

Sequence data were uploaded to the MG-RAST metagenomics analysis pipeline (version 4.03) for quality processing and basic taxonomic analysis.^20^ Low-quality regions (bases with quality scores <15) and reads shorter than 15 bp were discarded. Artificial replicate sequences and host-specific species sequences (eg, plant, human, or mouse) were also removed. Taxonomic abundance profiles at species level were generated by annotation against the RefSeq database housed within MG-RAST with the threshold of 95% identity^21^ using representative hit strategy. Abundance profiles for *P gingivalis, A actinomycetemcomitans,* and a control species, *Filifactor alocis*, were selected and extracted for analysis. We selected *F alocis* because it is a periodontal pathogen with no known ability to induce citrullination.

### Statistical Analysis

Descriptive statistics were used for the analysis of demographic and periodontal characteristics. For continuous data, median and interquartile range (IQR) were presented and Kruskal-Wallis tests were used to compare demographic and periodontal parameters (including PISA) between participant groups. Where significant differences were found for a periodontal parameter, post hoc testing was then performed using Bonferroni correction with a Dunn test. For categorical data (ie, clinical periodontal classification), χ^2^, or Fisher exact test where appropriate, was used to compare participant groups. The statistical significance level was set at .05 using 2-sided tests. Spearman ρ correlation was used to assess for associations.

For the analysis of the 3 periodontal pathogens, raw count data of each species (*P gingivalis, A actinomycetemcomitans, *and* F alocis*) were normalized and compared between different groups using the DESeq2 package (parameters: test = “Wald” and fitType = “parametric”) in R statistical software version 3.5.1 (R Project for Statistical Computing).^22^ *P* values were adjusted for multiple testing using false-discovery rate correction. A pseudocount of 0.5 was added to the normalized count to allow for log scale plotting. Effect sizes and standard errors were calculated using DESeq2 based on the negative binomial (gamma-Poisson) distribution.

## Results

### Baseline Characteristics

Forty-eight CCP+ at-risk individuals (mean [SD] age, 51.9 [11.4] years; 31 [65%] female), 26 patients with ERA (mean [SD] age, 54.4 [16.7] years; 14 [54%] female), and 32 healthy individuals (mean [SD] age, 49.4 [15.3] years; 19 [59%] female) were included. Groups were balanced for age, sex, and smoking status (Table 1). Of 26 patients with ERA, 10 (38%) were DMARD naive. Patients with ERA who had commenced DMARD therapy were all receiving monotherapy with a median duration of only 2 weeks; 1 patient had commenced sulfasalazine and the remainder, methotrexate. No CCP+ at-risk individuals or healthy individuals had received DMARDs.

**Table 1.** Baseline Characteristics of Study Participants According to RA Status^a^

Variable	No. (%)		
	Healthy Controls (n = 32)	CCP+ At-Risk (n = 48)	Early RA (n = 26)
Anti-CCP positive	0	44 (92)	25 (96)
Age, mean (SD), y	49.4 (15.3)	51.9 (11.4)	54.4 (16.7)
Female	19 (59)	31 (65)	14 (54)
Current smoker	6 (19)	12 (25)	4 (15)
Former smoker	12 (38)	19 (40)	13 (50)
Current disease-modifying antirheumatic drugs	0	0	16 (62)

Abbreviations: CCP, cyclic citrullinated peptide; CCP+, positive for CCP; RA, rheumatoid arthritis.

^a^ Groups were balanced for age, sex, and smoking status.

All CCP+ at-risk individuals and patients with ERA had tested positive for serum IgG anti-CCP2 antibodies using the Bioplex 2200 kit (BioRad) (titer ≥3 × upper limit of normal range) when they were recruited to the study. Of these 83 participants, 5 (6%) had a negative serum IgG anti-CCP2 test result using the Immunocap kit (Phadia) (Table 1) at the time of periodontal assessment.

### Periodontal Assessment

The percentage of periodontal sites with CAL 2 mm or greater, PPD 4 mm or greater, BOP, PDD, and active PDD were all greater in anti-CCP+ at-risk individuals compared with healthy individuals (Table 2). In contrast, there were no differences in any of these parameters between anti-CCP+ at-risk individuals and patients with ERA. The number of missing teeth was higher in patients with ERA compared with healthy individuals, likely reflecting the higher mean age in this group (54 and 49 years, respectively).

**Table 2.** Periodontal Assessments According to RA Status

Variable	Median (IQR)			*P* Value^a^
	Healthy Controls (n = 32)	CCP+ At-Risk (n = 48)	Early RA (n = 26)	
Missing teeth, No.	5 (3-8.5)	6 (4-14.5)	12 (5-24)^b^	.03
Sites with disease, %				
Clinical attachment level ≥2 mm	9 (3-18.1)	15.1 (6.3-51.3)	25.0 (7.1-64.7)	.06
Pocket depth ≥4 mm	0.6 (0-5.8)	11.4 (1.9-18.8)^c^	7.2 (0-20.1)	.005
Bleeding on probing	8.6 (1.9-16.7)	30.0 (8.6-46.3)^c^	19.0 (6.8-37.8)	.007
Periodontal disease^d^	0 (0-0.7)	3.3 (0-11.3)^c^	1.1 (0-13.1)	.001
Active periodontal disease^d^	0 (0-0.6)	1.9 (0-5.8)^c^	1.1 (0-6.6)	.001

Abbreviations: CCP+, positive for cyclic citrullinated peptide; IQR, interquartile range; RA, rheumatoid arthritis.

^a^ *P* value indicates comparison across all 3 groups (Kruskal-Wallis tests).

^b^ For significant results, Bonferroni correction with Dunn post hoc pairwise comparison is shown: early RA vs healthy controls, *P* = .01.

^c^ For significant results, Bonferroni correction with Dunn post hoc pairwise comparison is shown: CCP+ at-risk vs healthy controls, *P* = .004 for percentage of sites with pocket depth 4 mm or greater, *P* = .002 for percentage of sites with bleeding on probing, *P* = .01 for percentage of sites with periodontal disease, and *P* = .005 for percentage of sites with active periodontal disease.

^d^ We defined periodontal disease as clinical attachment level 2 mm or greater and pocket depth 4 mm or greater at the same site.

Overall clinical periodontal status of all participants is shown in Table 3. Dentists classified 35 of 48 CCP+ at-risk individuals (73%) as having periodontitis, compared with 12 of 32 healthy individuals (38%) (difference, 35%; 95% CI, 13%-53%; *P* = .02). The median (interquartile range) percentage of periodontal sites with disease was greater in CCP+ at-risk individuals compared with healthy individuals (3.3% [0%-11.3%] vs 0% [0%-0.7%]). Rates of periodontitis did not show difference in prevalence between CCP+ at-risk participants and patients with ERA (1.1% [0%-13.1%]) (difference, 19%; 95% CI, −3% to 40%; *P* = .10).

**Table 3.** Overall Clinical Periodontal Status According to Rheumatoid Arthritis Status

Patient Group	Periodontal Status, No. (%)		
	Healthy^a^	Gingivitis^b^	Periodontitis^c^
Healthy controls (n = 32)	10 (31)	10 (31)	12 (38)
Anti–cyclic citrullinated peptide–positive at-risk (n = 48)	2 (4)	11 (23)	35 (73)^d^
Early rheumatoid arthritis (n = 26)	3 (12)	9 (35)	14 (54)
Total (N = 106)	15 (14)	30 (28)	61 (58)

^a^ No periodontitis or gingivitis.

^b^ Gingivitis without periodontitis.

^c^ Periodontitis with or without gingivitis.

^d^ Anti–cyclic citrullinated peptide–positive at-risk vs healthy controls, *P* < .001 (Fisher exact test).

### Periodontal Inflamed Surface Area

Median (IQR) PISA in CCP+ at-risk individuals was 221 mm^2^ (81-504 mm^2^) compared with 40 mm^2^ (12-205 mm^2^) in healthy individuals and 116 mm^2^ (25-269 mm^2^) in patients with ERA (*P* = .006; Kruskal-Wallis test), indicating higher total periodontal inflammation in CCP+ at-risk individuals compared with healthy participants (*P* = .002) (eFigure in the Supplement).

### Ultrasonographic Assessment

All CCP+ at-risk individuals underwent US assessment. Synovitis was defined as the presence of gray scale synovial hypertrophy greater than or equal to 1 and PD signal greater than or equal to 1 (gray scale ≥1 and PD ≥1) at the same joint. Of 48 CCP+ at-risk individuals, 46 (96%) had no US synovitis, suggesting the absence of both clinical and subclinical joint inflammation in these subjects. Of the 2 patients who had US synovitis, 1 had synovitis in both first metatarsophalangeal joints and the other in both wrist joints.

### Three Periodontal Pathogens Selected From the Shotgun Metagenomes

The abundance of *P gingivalis*, *A actinomycetemcomitans,* and *F alocis* at healthy and diseased periodontal sites was compared according to RA status. Read counts of *P gingivalis*, *A actinomycetemcomitans,* and *F alocis* were generated from MG-RAST (RefSeq database with 95% identity) and normalized by DESeq2. At healthy periodontal sites, there was a greater abundance of *P gingivalis* in CCP+ at-risk individuals compared with healthy participants (effect size = 3.00; 95% CI, 1.71-4.29; *P* < .001 [Wald test]) and patients with ERA (effect size = 2.14; 95% CI, 0.77-3.52; *P* = .002 [Wald test]) (Figure). Interestingly, at diseased periodontal sites, both CCP+ at-risk and healthy participants had a greater abundance of *P gingivalis* compared with patients with ERA (effect size = 5.12; 95% CI, 3.34-6.90 and 4.60; 95% CI, 2.54-6.66; *P* < .001, respectively). There were no differences in the abundance of *A actinomycetemcomitans* or *F alocis* according to RA status at either healthy or diseased periodontal sites.

**Figure.**
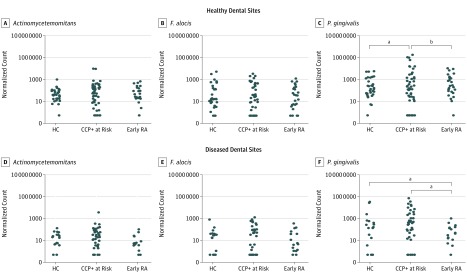
Abundance of Periodontal Bacteria at Diseased and Healthy Dental Sites According to Rheumatoid Arthritis (RA) Status The abundance of *Aggregatibacter actinomycetemcomitans*, *Filifactor alocis,* and *Porphyromonas gingivalis* compared between groups using the Wald test. CCP+ indicates individuals positive for anti–cyclic citrullinated peptide antibodies; HC, healthy control. ^a^Adjusted *P* value <.001. ^b^Adjusted *P* value <.05.

## Discussion

In this exploratory cross-sectional study, we have identified an increased prevalence of periodontal disease and periodontal inflammation in CCP+ at-risk individuals without joint inflammation. Furthermore, we identified an increased abundance of the periodontopathic bacterium *P gingivalis* at the healthy periodontal sites of CCP+ at-risk participants compared with control individuals and patients with ERA.

Several observational studies^7,8,23,24,25,26,27,28,29,30^ and a recent meta-analysis^6^ have confirmed an association between periodontal disease and established RA. However, whether or not periodontal disease is truly a trigger for inflammatory arthritis cannot be assessed in patients who have already developed RA. In the current study, we performed comprehensive periodontal examination, using quantitative periodontal measurement, overall clinical periodontal status, and total periodontal inflammatory burden (by PISA) in at-risk participants with anti-CCP antibodies but no clinical or subclinical synovitis. We found an increased prevalence of periodontal disease sites and overall clinical periodontitis as well as an increased PISA in CCP+ at-risk individuals compared with control individuals matched for age and smoking status. The presence of high-titer serum ACPA in these individuals indicates a break in tolerance and the development of RA-related systemic autoimmunity. Prospective data from the CCP+ at-risk cohort suggests that while not all these individuals can be considered to be pre-RA, some will go on to develop clinical arthritis and RA.^15^ This suggests periodontal inflammation may precede joint inflammation in the development of RA and supports the concept of the periodontium as a mucosal site of disease initiation. These findings are in line with a recent study^31^ showing a higher frequency and greater severity of periodontal disease in first-degree relatives of patients with RA, although in that study, only 9% of first-degree relatives with periodontitis were anti-CCP positive.

The microbiome in periodontal disease is distinct from that seen in periodontal health, with an increased abundance of pathogenic bacterial communities.^32,33^ *Porphyromonas gingivalis* is a key component of the so-called red complex of gram-negative bacteria associated with periodontal disease.^9^ It has been hypothesized that *P gingivalis*, through posttranslational citrullination of periodontal mucosal proteins, may provide an antigenic source for ACPA in RA.^11^ A recent study^34^ showed oral priming with *P gingivalis* can trigger an erosive ACPA-positive arthritis in an in vivo animal model. In the current study, we found an increased abundance of *P gingivalis* in subgingival plaque from the healthy periodontal sites of CCP+ at-risk participants compared with healthy individuals and patients with ERA. This association was not seen at diseased periodontal sites where *P gingivalis* was identified at similar levels in CCP+ at-risk and healthy participants. The reason for the lower abundance of *P gingivalis* at diseased sites in patients with ERA compared with the other groups is not fully clear, but this may be due to an early effect of therapy; 62% of these patients were receiving DMARDs and most had also received corticosteroids. It is possible that these treatments may have had an early influence on the periodontal microbiome. As periodontitis may be caused by a dysregulated inflammatory response initiated by the biofilm, it is possible that immunomodulatory therapies could have a direct effect on periodontal inflammation, with consequent effects on the microbiome. Also, DMARDs are believed to have antimicrobial properties.^35,36,37^ It is possible that *P gingivalis* may be affected in this way, and this would be an interesting area to explore in future work.

Interestingly, a recent study found evidence of dysbiosis of the subgingival microbiome in periodontally healthy patients with RA compared with controls, suggesting RA may be associated with changes to the periodontal microbiome independently of periodontal inflammation.^38^ Abundance of *P gingivalis* was no different between groups in that study, which may be expected, as the patients did not have periodontitis. In contrast, in the current study, *P gingivalis* was enriched at the periodontally healthy sites of CCP+ at-risk participants, the majority of whom also had periodontal disease sites. It is possible that the dysbiosis related to periodontitis has a broader effect specifically in CCP+ at-risk individuals, leading to dysbiosis at distant healthy periodontal sites. Of note, this association was not seen for *A actinomycetemcomitans* or *F alocis,* suggesting *P gingivalis* may be especially significant in CCP+ at risk. While *A actinomycetemcomitans* has been shown to be capable of citrullination,^12^ it is particularly associated with aggressive forms of periodontitis that were not seen in our participants.^39^

### Limitations

This study is limited by a relatively small sample size; CCP+ at-risk individuals are difficult to identify and our patients have been recruited from a national primary care study. Owing to frequency matching during the recruitment period, the final group numbers were unequal; despite the planned number of participants being exceeded, fewer patients were identified and recruited in the healthy control group and ERA group compared with the CCP+ at-risk group. Some individuals from all groups declined study participation. Unfortunately, details of eligible participants who declined in each group are not available. It is possible that this self-selection may have introduced bias. However, a participant survey conducted prior to periodontal assessment suggests no difference in access to dental care or self-reported oral symptoms between CCP+ at-risk and healthy control groups (eAppendix 3 and the eTable in the Supplement). Although participant groups were matched for smoking status, we did not match for diabetes, which is also associated with periodontal disease. However, the prevalence of diabetes in our participants was low and unlikely to have influenced the data (eAppendix 4 in the Supplement). We acknowledge that other rare systemic conditions are associated with periodontal disease (eg, neutrophil disorders, epidermolysis bullosa, Ehlers-Danlos syndrome, hematological malignant neoplasms).^40^ We were not aware of our participants being affected by these conditions, but they were not specifically excluded. These limitations mean that the findings of this study must be considered as exploratory. These data should be validated in other cohorts, and longitudinal follow-up will be important to assess whether periodontal disease predicts the onset of clinical arthritis in CCP+ at-risk individuals.

## Conclusions

This study is the first, to our knowledge, to demonstrate an increased prevalence of periodontal disease together with an increased abundance of *P gingivalis* in anti-CCP–positive individuals at risk of RA. These data suggest periodontal inflammation and the enrichment of *P gingivalis* may precede joint inflammation in RA and support an association between these risk factors and disease initiation. This study adds to an emerging evidence base linking periodontal and systemic disease and, therefore, further highlights the potential importance of improving dental health and reducing the burden of periodontal disease on the risk of chronic systemic diseases such as RA. Importantly, these findings suggest periodontal inflammation may be a legitimate target to explore for preventive intervention in RA.

## Notes

Supplement. **eAppendix 1.** Ultrasound Assessment **eAppendix 2.** Subgingival Plaque Collection and DNA Extraction **eFigure.** Periodontal Inflamed Surface Area (PISA) in mm^2^ According to RA Status **eAppendix 3.** Participant Survey **eAppendix 4.** Comorbidities **eTable. **Patient-Reported Data on Periodontal Disease
