# Supplementary material for: Prevalence of Periodontal Disease and Periodontopathic Bacteria in Anti–Cyclic Citrullinated Protein Antibody–Positive At-Risk Adults Without Arthritis
Source: JAMA Netw Open. 2019 Jun 7;2(6):e195394. doi: 10.1001/jamanetworkopen.2019.5394 (PMC6563551; doi:10.1001/jamanetworkopen.2019.5394)
Supplement: Supplement. — eAppendix 1. Ultrasound Assessment eAppendix 2. Subgingival Plaque Collection and DNA Extraction eFigure. Periodontal Inflamed Surface Area (PISA) in mm2 According to RA Status eAppendix 3. Participant Survey eAppendix 4. Comorbidities eTable. Patient-Reported Data on Periodontal Disease [file jamanetwopen-2-e195394-s001.pdf]

Supplementary Online Content

Mankia K, Cheng Z, Do T, et al. Prevalence of periodontal disease and periodontopathic bacteria in anti–cyclic citrullinated protein antibody–positive at-risk adults without arthritis. *JAMA Netw Open*. 2019;2(6):e195394. doi:10.1001/jamanetworkopen.2019.5394

eAppendix 1. Ultrasound Assessment

eAppendix 2. Subgingival Plaque Collection and DNA Extraction

eFigure. Periodontal Inflamed Surface Area (PISA) in mm^2 According to RA Status

eAppendix 3. Participant Survey

eAppendix 4. Comorbidities

eTable. Patient-Reported Data on Periodontal Disease

This supplementary material has been provided by the authors to give readers additional information about their work.

© 2019 Mankia K et al. *JAMA Network Open.*

Online supplemental materials

eAppendix 1. Ultrasound assessment

A standardised 38-joint ultrasound (US) protocol was performed on all CCP+ at-risk subjects by two experienced expert MSK sonographers (JLN, LH): wrists, metacarpophalangeal joints (MCPJs), proximal interphalangeal joints (PIPJs), elbows, knees, ankles and metatarsophalangeal joints (MTPJs) were scanned bilaterally in longitudinal and transverse planes, dorsally. In addition, other joint regions could be scanned if symptomatic.

eAppendix2. Subgingival plaque collection and DNA extraction

Two sterile paperpoints (Maillefer Pro Taper Paper Points F3; The Dental Directory, UK) were used to collect subgingival plaque from each site, avoiding bleeding, and immediately placed in a cryovial containing 0.5ml of RNA protect Bacteria Reagent (Qiagen, Germany). Cryovials were vortexed and centrifuged at 8000g for 1 minute. Supernatants were removed by pipetting and cryotubes containing pellets were stored at -80°C.

To increase the yields of DNA extraction, the paper-points carrying the plaque were thoroughly vortexed in the Micro-bead solution from the kit to re-suspend the bacterial cells and the samples were then incubated with lysozyme (10 mg/mL) at 37 °C for 10 minutes to break the cell walls. Then the extraction continued with combined chemical and mechanical lysis as described in the kit protocol.

© 2019 Mankia K et al. *JAMA Network Open.*

eFigure: Periodontal inflamed surface area (PISA) in mm^2 according to RA status. PISA was higher in CCP+ at-risk compared with HC and ERA (p=0.0055). Red cross-hairs indicate median values. HC, healthy controls; CCP+ at-risk, anti-CCP positive at-risk individuals; ERA, early RA patients.

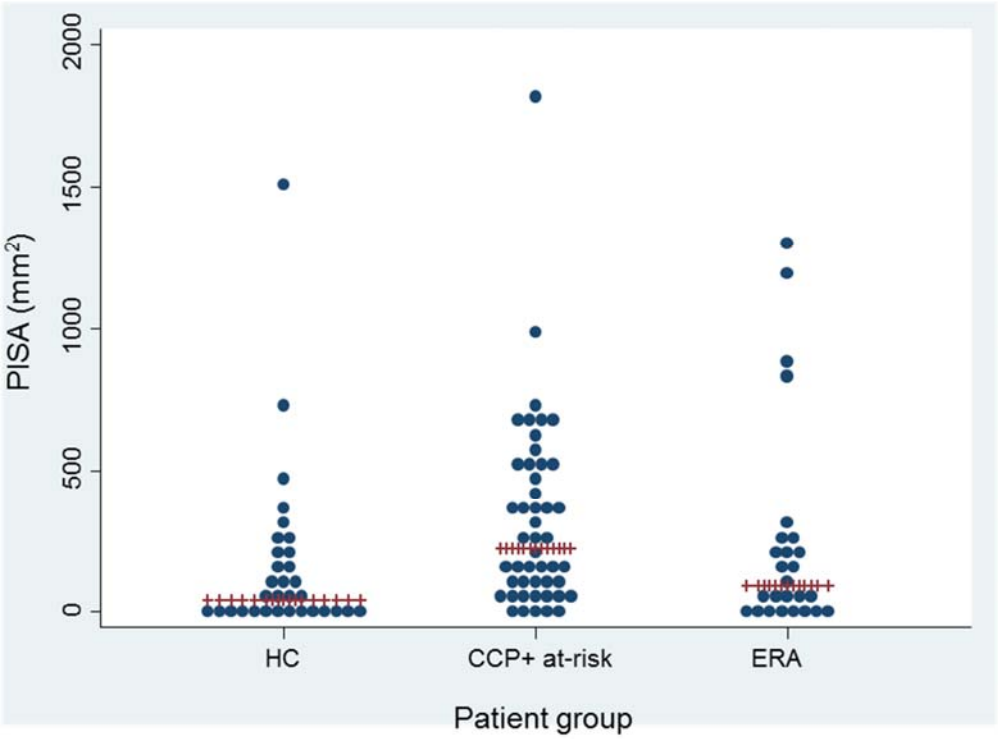

eAppendix 3. Participant survey

Prior to the periodontal assessment participants were given a questionnaire on oral symptoms, access to dental care and previous dental treatments. The results (Etable 1) show similar oral symptoms and dental treatment between participant groups.

eAppendix 4. Comorbidities

All participants completed a questionnaire about pre-specified co-morbid medical conditions prior to the periodontal examination. 4/48 (8%) CCP+ at-risk, 0/32 (0%) HC and 1/26 (4%) ERA reported they had diabetes mellitus. There was no significant difference in the proportion of participants with diabetes mellitus between groups (Fisher exact test, p=0.20)

© 2019 Mankia K et al. *JAMA Network Open.*

eTable. Patient-reported data on periodontal disease. P value indicates comparison between groups (Fisher's exact test). Significant P values (p<0.05) are highlighted in bold text.

		**Healthy controls (n=32) n(%)**	**CCP+ at-risk (n=47) n(%)**	**ERA (n=26) n(%)**	**P**
Have you had these oral conditions?					
Bleeding gums					
	≤ 2 years ago	10 (31)	24 (51)	11 (42)	0.22
	Ever	9 (28)	9 (19)	6 (23)	0.65
	Never	12 (38)	12 (26)	4 (15)	0.16
	Don't know	1 (3)	2 (4)	5 (19)	**0.05**
Sore + swollen gums					
	≤ 2 years ago	10 (31)	15 (32)	1 (4)	**0.002**
	Ever	3 (9)	7 (15)	6 (23)	0.29
	Never	17 (53)	19 (40)	16 (62)	0.20
	Don't know	2 (6)	6 (13)	3 (12)	0.71
Loose teeth					
	≤ 2 years ago	4 (13)	11 (23)	3 (12)	0.38
	Ever	4 (13)	8 (17)	4 (15)	0.94
	Never	19 (59)	23 (49)	18 (69)	0.77
	Don't know	5 (16)	5 (11)	1 (4)	0.33
Toothache					
	≤ 2 years ago	10 (31)	23 (49)	6 (23)	0.06
	Ever	3 (9)	13 (28)	12 (46)	**0.006**
	Never	17 (53)	9 (19)	4 (15)	**0.002**
	Don't know	2 (6)	2 (4)	4 (15)	0.28
Chronic bad breath					
	≤ 2 years ago	3 (9)	5 (11)	2 (8)	1.00
	Ever	1 (3)	3 (6)	2 (8)	0.76
	Never	21 (66)	29 (62)	18 (69)	0.84
	Don't know	7 (22)	10 (21)	4 (15)	0.86
Do you go to the dentist regularly? (Yes)		26 (81)	36 (77)	26 (100)	**0.01**
When was your last dental examination?					
	<1 year ago	17 (53)	29 (62)	14 (54)	0.70
	1-3 years ago	5 (16)	6 (13)	2 (8)	0.64
	>3 years ago	1 (3)	3 (6)	6 (23)	**0.04**
	Don't know	9 (28)	9 (19)	4 (15)	0.48
Ever told to have gum disease? (Yes)		4/31 (13)	13 (28)	5 (19)	0.28
Ever had gum treatment? (Yes)		2 (6)	4/43 (9)	3 (12)	0.90
Lost any of your natural teeth? (Yes)		21 (66)	37 (79)	18 (69)	0.43
If so, did you lose any teeth because of gum disease? (Yes)		0/18 (0)	5/37 (14)	1 (4)	0.25
Do you have any gum problems? (Yes)		8 (25)	15/43 (35)	4/25 (16)	0.24

© 2019 Mankia K et al. *JAMA Network Open.*
